# The prevalence of long-term rehabilitation following motor-vehicle crashes in Saudi Arabia: a multicenter study

**DOI:** 10.1186/s12891-022-05153-8

**Published:** 2022-03-03

**Authors:** Suliman Alghnam, Mashael Alghamdi, Sarah Alzahrani, Sufyan Alzomai, Abdullah Alghannam, Ibrahim Albabtain, Khalid Alsheikh, Miasem Bajowaiber, Ali Alghamdi, Fatimah Alibrahim, Omar Aldibasi

**Affiliations:** 1 Population Health Department, King Abdullah International Medical Research Center (KAIMRC), King Saud bin Abdulaziz University for Health Sciences, Riyadh, Saudi Arabia; 2 Lifestyle and Health Research Center, Health Sciences Research Center , Princess Nourah bint Abdulrahman University, Riyadh, Saudi Arabia; 3 Department of Surgery, King Abdulaziz Medical City (KAMC), Riyadh, Saudi Arabia; 4 College of Medicine, King Saud bin Abdulaziz University for Health Sciences, Riyadh, Saudi Arabia; 5 Department of Orthopedics, King Abdulaziz Medical City (KAMC), Riyadh, Saudi Arabia; 6 National Center for Road Safety, Ministry of Transportation, Riyadh, Saudi Arabia; 7 College of Medicine, King Saud University, Riyadh, Saudi Arabia; 8 Biostatistics Department, King Abdullah International Medical Research Center (KAIMRC), King Saud bin Abdulaziz University for Health Sciences, Riyadh, Saudi Arabia

**Keywords:** Rehabilitation, MVC, Polytruma, ISS, GCS

## Abstract

**Introduction:**

In Saudi Arabia, motor-vehicle crashes (MVC) are the leading cause of disability-adjusted life years (DALYs). There is limited information locally on the magnitude and need for rehabilitation following MVC. This study examined the prevalence of MVC patients requiring long-term rehabilitation and the epidemiology of associated injuries.

**Methods:**

A retrospective study was conducted at four hospitals of the National Guard Hospitals Affairs from January 2016 to March 2019. The study used data from an institutional trauma registry of all MVC admissions. Chi-square tests, bivariate and multivariate analyses were conducted to compare patients requiring long-term and short-term rehabilitation.

**Results:**

The study included 506 patients. The study population was relatively young, with an average age was 32.8 ± 15.5 years, and the majority were males. Over two-thirds (71.3%) of patients required long-term rehabilitation. Half the patients sustained multiple fractures, and 17.0% sustained traumatic brain injuries. Overall, 53.1 and 61.8% of patients required occupational and physiotherapy, respectively. Those admitted to the intensive care unit were four times more likely to need long-term rehabilitation.

**Conclusions:**

We found a significant burden of long-term rehabilitation following MVC. Patients were relatively young, thus posing a significant burden on future healthcare utilization. Policymakers should use these findings to guide primary, secondary, and tertiary prevention to improve health outcomes.

## Introduction

Motor-vehicle crashes (MVC) are an alarming issue globally [1]. According to the World Health Organization (WHO), an estimated 1.35 million die yearly due to MVC [2]. One of the Sustainable Development Goals was to reduce the global MVC death and injuries by 50% by 2020 [2]. In addition to the health burden of MVCs, their economic burden is as high as $518 billion worldwide and $65 billion in low-and middle-income countries [3].

MVCs are one of the leading causes of injury-related disability, accounting for one-fourth of all injury-related disabilities in the United States [4]. Many MVC injuries may be severe and lead to temporary or permanent disabilities and this burden may accumulate over time [5]. A study of developing countries found that a significant increase in the frequency of MVC (38.6%) is associated with spinal cord injury (SCI) [6].

In Saudi Arabia, MVCs are the leading cause of injuries, responsible for 52% of all injuries [7]. This figure is five times higher than the global average of 12% [8]. MVCs are also the leading cause of disability-adjusted life years (DALYs) lost in 2017 (9.2%) [9]. Over the past two decades, Saudi has documented 86,000 fatalities and 611,000 injuries in MVC [8].

Disabilities associated with MVCs can be defined as any reduction in levels of functioning due to injury [10]. Examples of these impairments are physical and cognitive limitations due to neurotrauma paralysis, partial or complete amputation of the limbs, limb deformation resulting in mobility impairments, psychological trauma, and sensory disability such as blindness or deafness [11].

While the burden is high, data on the disabilities and burden of nonfatal MVC is limited. A review of the literature on MVC found that the most common injuries were head, neck, and limbs [8]. These injuries may lead to various sequalae, and depending on the severity, patients might require short to extensive long-term rehabilitation. Patients with traumatic brain injuries, in particular, can have long debilitating effects that require long rehabilitation [12]. Consequently, patients may struggle with a physical disability, cognitive disorders, and social difficulties.

Rehabilitation services can include physical therapy and occupational therapy [13]. Patients who require rehabilitation services may benefit from relatively long-term and specialized rehabilitation care and are often referred to particular rehabilitation centers such as spinal, amputee, or neurorehabilitation [14]. However, without understanding the need for such services, the ability to plan and create rehabilitation pathways might be challenging.

One of the common consequences of MVC is polytrauma, defined as “the Abbreviated Injury Scale (AIS) ≥ 3 for at least two different body regions” [15]. Compared with non-polytrauma, patients suffering from polytrauma have significantly longer hospital length of stay (LOS) (20.0 days vs. 16.8 days, respectively) and longer intensive care unit (ICU) (10.1 days vs. 8.3 days, respectively) [15]. Understanding this issue among MVC patients is critical as it may predict the need for long-term rehabilitation.

There is limited information on the need for rehabilitation following MVC in Saudi Arabia. It is imperative to understand the current status to assess future needs for rehabilitation services locally. We aim to determine the prevalence of patients that require long-term rehabilitation. This study will provide a basis for the demand for rehabilitation services in Saudi Arabia by answering this research question. Furthermore, it will provide a basis to galvanize prevention efforts to promote traffic safety and ultimately improve population health.

## Methods

This was a retrospective study conducted at four National Guard Hospitals Affairs (NGHA) hospitals in Saudi Arabia from January 2016 to March 2019. All patients meeting the inclusion criteria were identified. Data captured included four locations: King Abdulaziz Medical City in Riyadh with 394 patients, King Abdulaziz Medical City in Jeddah total number 63, King Abdulaziz Hospital in Al-Ahsa with 39 patients, Al-Imam Abdulrahman bin Faisal Hospital in Dammam included ten patients. Data were ascertained from the trauma registry provided comprehensive information on trauma patients who were admitted to the hospital for at least one day following MVC during the study period.

Next, rehabilitation data were ascertained from the electronic medical records. The study population comprised all patients ≥16 years old who were admitted to KAMC due to MVC between 2016 and 2019. A trained research coordinator reviewed all admitted patients with MVC and verified patients were actually injured in MVC and hospitalized. Patients treated in the ED then immediately discharged were excluded from the study.

Patients were classified as requiring long-term rehabilitation if they were diagnosed with one of the following injuries: spinal cord injury, amputation, traumatic brain injury, lower extremity fracture, multiple fractures, open fracture or polytrauma. This was based on two physicians’ expert opinions and consensus (an orthopedic surgeon and a rehabilitation physiatrist). In addition, long-term rehabilitation users included those discharged and seeking care in outpatient clinics for at least three months. Patients who required short-term rehabilitation were those who sought rehabilitation services for three months or less.

The trauma registry includes demographics, injury type and cause, severity, LOS, and discharge status. To complement information not available in the registry, research coordinators used a pre-designed data collection sheet that includes access to rehabilitation, rehabilitation length of inpatient stay, description of patient’s disability, date of discharge, and discharge status or death. A patient was considered disabled if they suffered from a long-term impairment of function [4]. This includes if the patient suffered any amputation, fracture, traumatic brain injury, paralysis, and limb deformation [6].

Patients were classified based on the site and the severity of the injury. The injuries were classified using the AIS in all five major body regions into minor (1 point), up to severe or maximal (untreatable) (6 points) [16]. Only the three highest AIS scores in each body region are used to calculate the Injury Severity Score (ISS), which ranges between 0 and 75. A higher score indicates a more severe injury (1–8 minor, 9–15 moderate, 16–24 severe, 25–75 very severe) [17]. The hospital LOS was the time from ER admission until hospital discharge. The study was retrospective a chart review without any direct contact with patients and was performed in accordance with the Declarations of Helsinki. The Institutional Review Board (IRB) of King Abdulah International Medical Research Center (KAIMRC) reviewed the protocol, waived informed consent, and approved the study (number: 20/103/R).

### Statistical analysis

The trauma registry in KAMC includes over 20,000 patients. In this study, the sample size was calculated using 95% as a confidence interval, 5% margin of error, and assuming *p* = 0.5, the precision of 5% of the true value. The minimum required sample size is 385 patients to estimate the prevalence [18]. We included all patients in the trauma registry who met the inclusion criteria for a total of 506 patients.

Descriptive statistics were carried out in the form of mean, standard deviation (SD), and frequencies. The outcome is the prevalence of MVC patients who require long-term rehabilitation. It was defined as the proportion of patients with long-term rehabilitation as part of the overall admitted MVC patients. We also estimated the prevalence of patients who received interdisciplinary rehabilitation, defined as receiving all the services including physiotherapy, occupational therapy, psychiatric treatment, and social service support. For inferential statistics, chi-square tests were applied to compare long-term and short-term rehabilitation patients. Continuous variables, including ISS score and LOS, were compared using Student’s t-test. In addition, multivariate logistic regression techniques were used to model the probability of long-term rehabilitation with associated variables. We used multivariate forward selection regression with entry criteria of at least a *p*-value 0.3 and retained variables at a p-value of 0.05. The following variables were entered into the initial model: age, gender, BMI, mechanism of injury, body region, surgery, and ICU admission. The model included the following variables: ICU admission (“no” as the reference group), surgery (“no” as the reference group), and affected body region (“abdomen” as the reference, head and neck, face, thorax, extremities, and external and other). Statistical analyses were performed using STATA version 15.0 software, and a p-value of < 0.05 was declared as statistically significant.

## Results

A total of 506 medical records were retrieved from the trauma registry upon meeting the inclusion criteria. The mean (SD) age of patients was 32.8 (15.5) years, and a majority were males (83.3%). Patients were relatively young (44.0% between the ages of 17–25, Table 1). The most common injury mechanism was occupant or driver in an MVC, followed by motorcycle and pedestrian injuries. The most common site of injury is extremities (41.6%) followed by head and neck (20.4%) and thorax (13.1%).

**Table 1:** Characteristics of the patients admitted following MVC by rehabilitation status (*n* = 506)

Variables	Total	Short-term Rehab	Long-term Rehab	***p***-value
		N (%) Mean (±SD)	N (%) Mean (±SD)	
**Number of patients**	506	145 (28.66)	361 (71.34)	
**Age**	32.83 (±15.54)	34.73 (±15.56)	32.07 (±15.49)	0.08
**Age groups (Years)**				0.05
17–25	223 (44.07)	50 (22.42)	173 (77.58)	
26–45	187 (36.95)	64 (34.22)	123 (65.78)	
46–64	68 (13.43)	23 (33.82)	45 (66.18)	
> = 65	28 (5.53)	8 (28.57)	20 (71.43)	
**Gender**				0.06
Male	422 (83.39)	114 (27.01)	308 (72.99)	
Female	84 (16.60)	31 (36.9)	53 (63.1)	
**Mechanism of Injury**				0.48
MVC	394 (77.86)	109 (22.66)	285 (72.34)	
Pedestrian	64 (9.09)	13 (28.26)	33 (71.74)	
Motorcycle	66 (13.04)	23 (34.85)	43 (65.15)	
**Site of injury**				0.02*
Head & Neck	103 (20.43)	27(26.21)	76(73.79)	
Face	42 (8.33)	12(28.57)	30(71.43)	
Thorax	66 (13.09)	18(27.27)	48(72.73)	
Abdomen	32 (6.34)	17(53.13)	15(46.88)	
Extremities	210 (41.66)	51(24.29)	159(75.71)	
External and others	51 (10.12)	18(35.29)	33(64.71)	
**Rehabilitation Facilities**				
**Discharge reffereal to Rehabilitation**				0.01*
No	167 (33.2)	91(54.49)	76 (45.51)	
Yes	336 (66.79)	54 (16.07)	282 (83.93)	
**Occupational Therapy**				0.01*
No	237 (46.83)	112(47.26)	125(52.74)	
Yes	269 (53.16)	33(12.27)	236(87.73)	
**Physiotherapy Therapy**				0.01*
No	193 (38.14)	100(51.81)	93(48.19)	
Yes	313 (61.86)	45(14.38)	268(85.62)	
**Psychiatric treatment**				0.01*
No	479 (94.66)	143(29.85)	336(70.15)	
Yes	27 (5.34)	2(7.41)	25(92.59)	
**Social services**				0.01*
No	381 (75.3)	132(34.65)	249(65.35)	
Yes	125 (24.7)	13(10.4)	112(89.6)	
**Interdisciplinary Rehabilitation**				0.01*
No	473(93.47)	143(30.23)	330(69.77)	
Yes	30(5.92)	2(6.67)	28(93.33)	
**Required surgery**				0.01*
No	182 (35.96)	68 (37.36)	114 (62.64)	
Yes	324 (64.03)	77 (23.77)	247 (76.23)	
**ICU admission**				0.01*
No	350 (69.16)	123 (35.14)	227 (64.86)	
Yes	156 (30.83)	22 (14.1)	134 (85.9)	
**Injury severity scale**	11.8 (±9.9)	5.54 (±4.97)	13.45 (±10.57)	0.01^
**Glasgow coma scale**	13.36(±0.15)	14.37 (±2.1)	12.95 (±3.67)	0.01^
**LOS in ICU (days)**	4.20(±0.44)	1.13 (±3.24)	5.47 (±10.86)	0.01^
**Length of rehabilitation (days)**	35.76(±3.68)	17.13 (±64.70)	43.25 (±87.74)	0.01^
**Discharge status**				0.017*
**Alive**	486 (80)	144 (29.63)	342 (70.37)	
**Dead**	20 (4)	1 (5)	19 (95)	

*LOS* length of stay, *ICU* intensive care unit

*p value≤0.05-stastically significant chi square

^*p* value≤0.05-stastically significant t-test

Based on the classification of long-term rehabilitation, 71.3% of patients required long-term rehabilitation. 336 (66.8%) of the injured population utilized any rehabilitation services. More than half (53.1%) utilized occupational therapy, and 61.9% accessed physiotherapy treatments, while 168 (33.2%) used neither service. Among patients, 4% died following their injuries. Multiple fracture disabilities were the most common among patient disabilities (50.6%), followed by a single fracture (33.4%) and polytrauma (18.4%, Table 2).

**Table 2:** The type of injury sustained among the patients population across rehabilitation duration

Type	Yes N (%)	No N (%)
**Amputation**	4 (0.79)	502 (99.20)
**Traumatic Brain Injury**	86 (16.99)	420 (83.00)
**Spinal Cord Injury**	22 (4.34)	484 (95.65)
**Single Fracture**	169 (33.39)	337 (66.60)
**Multiple Fracture**	256 (50.59)	249 (49.20)
**Complicated Fracture**	16 (3.16)	489 (96.64)
**Polytrauma**	93 (18.37)	413 (81.62)

In comparing patients who required long-term rehabilitation to patients who required short-term rehabilitation, there was a significant difference observed between the various age groups based on rehabilitation status (p-value = 0.04) Table 1. Furthermore, among those who required surgery, 76.2% needed long-term rehabilitation. We found that both undergoing surgery and ICU admission were significantly associated with longer rehabilitation duration Table 1.

Patients who needed long-term rehabilitation sustained more severe injuries than those who required short-term rehabilitation. This was represented by a significantly higher ISS (13.45) and lower GCS scores than in short-term rehabilitation. Longer LOS in the ICU was associated with patients’ rehabilitation status 5.47 (±10.86) for long-term rehabilitation vs. 1.13 (±3.24) days for the short-term rehabilitation group (Table 1).

Furthermore, we evaluated predictors of long-term rehabilitation using a multivariate logistic regression (Table 3). We found ICU admission to be associated with a fourfold increase in the likelihood of long-term rehabilitation with OR = 3.98 (95% CI = 2.32–6.83). In addition, patients who required surgery were 60% more likely to require long-term rehabilitation than those who did not undergo surgery (OR = 1.6; 95% CI = 1.07–2.69). Body regions were also associated with long-term rehabilitation. Injuries to the head and neck, face, thorax, or extremities were more likely to require long-term rehabilitation compared to abdomen injuries (OR = 2.92 (95% CI = 1.21–7.05), OR = 3.25 (95% CI = 1.18–8.96), OR = 2.94 (95% CI = 1.15–7.50), OR = 3.90 (95% CI = 1.70–8.93), respectively.

**Table 3:** Multivariate logistic regression analysis for long term rehabilitation

Variable	Odds Ratio	Lower 95% CI	Upper 95%CI
**ICU admission**			
No	Ref	Ref	Ref
Yes	3.979	2.317	6.834
**Patient required surgery**			
No	Ref	Ref	Ref
Yes	1.696	1.07	2.686
**Body region -**			
Abdomen	Ref	Ref	Ref
Head and Neck	2.925	1.212	7.055
Face	3.253	1.18	8.967
Thorax	2.942	1.154	7.5
Extremities	3.902	1.704	8.935
External and others	2.225	0.856	5.782

## Discussion

We found that the vast majority of MVC patients required long-term rehabilitation. Moreover, a large percentage of MVC patients were individuals of economically reproductive age (17–25 years old). These findings underline the burden of MVC on population health. Major investment in primary prevention by increasing traffic safety is desperately needed to address this burden. In addition, there is a need to improve and expand rehabilitative care services to meet the high demand represented by the increased demand due to injuries.

Reckless driving behaviors may explain the severity of MVC injuries found in our study. Local studies on MVC trends have shown that unsafe driving behaviors such as speeding, low seatbelt compliance, among other traffic law violations, are frequent contributors to the high incidence of MVC injuries [5]. Enforcement of traffic laws has improved compliance with safe driving [19]. Saher, an automated traffic violation detection system implemented in 2010, was associated with a 46% reduction in the likelihood of mortality and a 20% reduction of severe injuries in Riyadh [20]. Therefore, further investment in enforcement may reduce MVC and associated injuries.

The majority of patients admitted for MVC within the study timeframe were young and male. This might be because women were not legally permitted to drive in Saudi Arabia before 2018 [21]. Moreover, the high prevalence of MVC among young males is also consistent with global estimates of MVC [22]. Another reason for the frequency of younger patients may have to do with the age distribution of the Saudi population, where the median age is 25.7 years [23].

Furthermore, we found that motor-vehicle drivers and passengers are significantly more likely to need rehabilitation than motorcyclists and pedestrians. Comparatively, this was not the case in other countries. A study on the prevalence of MVC-related disability in Iran found that pedestrians and motorcyclists were 1.7 and 1.1 times more likely to become disabled, respectively [3]. One potential explanation is that pedestrians and motorcyclists are less likely to survive to be rehabilitated. According to a WHO report on road safety, it is estimated that almost half of the approximately 1.2 million people who die in MVC yearly are pedestrians, motorcyclists, and cyclists [24].

Head injuries pose a major threat to patients’ outcomes. A study estimated that head and neck injuries were responsible for 34% of MVC fatalities [25]. Significantly, nearly 80% of patients in our study population with head and neck injuries required long-term rehabilitation. Head injury is considered to be a significant risk factor for long-term disability [26]. Consequently, patients can suffer debilitating cognitive, behavioral, and emotional impairments that could develop into aggression, anxiety disorders, and other disabilities [26]. A cohort study involving 258 Saudi patients found 32.5% of head injury patients suffered long-term disabilities [5].

Recovery time is a major concern for patients and their families. It is usually difficult for healthcare providers to predict recovery time due to various influencing factors. The impact ISS and GCS play in need of long-term rehabilitation could guide clinicians’ expectations while delivering care and communicating with MVC patients. Considering the magnitude and prevalence of MVC in Saudi Arabia, access to rehabilitation must be improved, especially as motor vehicles remain the dominant mode of transportation [27]. Markedly, it is estimated that about one-third of all hospital beds in the Kingdom are occupied by MVC patients, posing a significant burden on the healthcare sector and national economy [5].

Investment in improving access and capacities of rehabilitation services is a necessary measure towards lowering the burden of MVC injuries, a primary objective of the Saudi Vision 2030. An issue to be considered is the limited number of specialized rehabilitation centers. Only two major specialized rehabilitation centers exist in the Riyadh region, serving a population exceeding 8 million individuals [28]. Limited access to rehabilitation services may result in a significant proportion of the population having reduced life quality.

This study offers a glance into how rehabilitation services are utilized by MVC patients and the epidemiology of MVC injuries locally. However, it is not without limitations. Since data were retrieved from one health system, we cannot say the findings are generalizable to the whole population. Another limitation is the retrospective nature of the study. Therefore, causality cannot be determined.

Moreover, we did not collect data about patients’ cognitive, behavioral and emotional complications or barriers to receiving rehabilitation services. A potential area for future research would be to explore rehabilitation access and capacity for MVC patients in the Kingdom. We recommend that more research in planning and executing primary preventative measures against MVC should be invested to reduce the burden of MVC.

## Conclusion

The study found a significant burden of long-term rehabilitation following MVC. Patients were relatively young and will pose a major burden on future healthcare utilization and population health. Policymakers can use these findings to guide primary, secondary, and tertiary prevention to reduce the burden of MVC and improve health outcomes.

## Data Availability

All data are available from the corresponding author upon reasonable request.
