# Correction to: The prevalence of long-term rehabilitation following motor-vehicle crashes in Saudi Arabia: a multicenter study

**DOI:** 10.1186/s12891-022-05286-w

**Published:** 2022-04-12

**Authors:** Suliman Alghnam, Mashael Alghamdi, Sarah Alzahrani, Sufyan Alzomai, Abdullah Alghannam, Ibrahim Albabtain, Khalid Alsheikh, Miasem Bajowaiber, Ali Alghamdi, Fatimah Alibrahim, Omar Aldibasi

**Affiliations:** 1 Population Health Department, King Abdullah International Medical Research Center (KAIMRC), King Saud bin Abdulaziz University for Health Sciences, Riyadh, Saudi Arabia; 2 Lifestyle and Health Research Center, Health Sciences Research Center, Princess Nourah bint Abdulrahman University, Riyadh, Saudi Arabia; 3 Department of Surgery, King Abdulaziz Medical City (KAMC), Riyadh, Saudi Arabia; 4 College of Medicine, King Saud bin Abdulaziz University for Health Sciences, Riyadh, Saudi Arabia; 5 Department of Orthopedics, King Abdulaziz Medical City (KAMC), Riyadh, Saudi Arabia; 6 National Center for Road Safety, Ministry of Transportation, Riyadh, Saudi Arabia; 7 College of Medicine, King Saud University, Riyadh, Saudi Arabia; 8 Biostatistics Department, King Abdullah International Medical Research Center (KAIMRC), King Saud bin Abdulaziz University for Health Sciences, Riyadh, Saudi Arabia

**Correction to: BMC Musculoskelet Disord 23, 202 (2022)**

**https://doi.org/10.1186/s12891-022-05153-8**

Following the publication of the original article [1] the authors noticed that author’s name has missing letter-L in Abdulah. The correct name is Abdullah F. Alghannam.

The original article [1] has been updated.
